# Co-creating innovative and accepted legume-based dishes for school canteens with adolescents in a low socioeconomic area

**DOI:** 10.1016/j.foodqual.2024.105343

**Published:** 2025-02

**Authors:** Margarita Kokkorou, Sara Spinelli, Caterina Dinnella, Lapo Pierguidi, Jan Wollgast, Petros Maragkoudakis, Erminio Monteleone

**Affiliations:** a European Commission, Joint Research Centre (JRC), Ispra, Italy; b Department of Agriculture, Food, Environment and Forestry, University of Florence, Florence, Italy

**Keywords:** Sensory properties, Emotions, Health, Vegetables, Food innovation, Food neophobia

## Abstract

The promotion of sustainable diets, that valorise and give stage to plant-based foods, is beneficial for both human and environmental health. Adolescence is crucial to support healthy and sustainable eating. Despite this, adolescents remain an understudied group, especially those with a lower economic status. This study aimed to develop innovative, sustainable and healthy legume-based dishes for school canteens through co-creation with adolescents in a low socioeconomic status area with high immigration rate.

Nineteen adolescents and four chefs participated in iterative sessions of focus group discussions using a combination of methods (Jobs-To-Be-Done, free association tasks, SCAMPER (Substitute-Combine-Adapt-Modify-Purpose-Eliminate-Rearrange) technique) to identify ideas of new legume-based dishes. Subsequently, 91 adolescents rated their willingness-to-try 28 dish concepts based on these ideas. Six concepts were selected and translated into prototypes that underwent further evaluation by 138 adolescents who assessed their liking, sensory and emotional responses.

Results showed that all dishes were well-received, with lower acceptance among high-neophobic adolescents, yet none of the selected dishes were rejected. Exploring individual differences in liking identified two clusters with different hedonic patterns. Emotional characterization of dishes showed those that elicit positive emotional responses high in arousal and novelty in all adolescents, independently from food neophobia, increasing their likelihood of acceptance.

The study shows that co-creation with adolescents, in combination with understanding individual differences, are promising strategies to develop innovative, healthy and well-received legume-based dishes for school canteens.

## Highlights

• Individual difference in Food Neophobia were considered in the co-creation process.
• Identifying emotional “Jobs -To -Be -Done” is vital in food co-creation.
• Validating co-created dishes requires studying individual liking/ emotion differences.
• Underrepresented vulnerable adolescents were involved in the co-creation process.

## 1 Introduction

### 1.1 Promoting legume consumption for health and sustainability

Diets, non-communicable diseases (NCDs) and environmental sustainability are closely linked. The rise in unhealthy diets and the high prevalence of NCDs are positively associated with the decline of planetary health (Branca et al., 2019). Unhealthy diets are among the modifiable risk factors of NCDs, including obesity, type 2 diabetes, cancers and cardiovascular diseases (Al-Jawaldeh and Abbass, 2022, WHO, 2022b). Adhering to healthy and sustainable diets could significantly reduce all-cause mortality and cancer burden, while promoting planetary health by reducing greenhouse gas emissions and land use (Laine et al., 2021). Legumes are key to healthy and sustainable diets, requiring minimal water while offering high nutritional value (FAO/OCCP, 2016). Their consumption is associated with a protective effect against NCDs, including cancer (DGE, 2016, NNR, 2023, WCRF/AICR, 2018), diabetes (NNR, 2023), cardiovascular disease (NNR, 2023) and coronary heart disease (DGE, 2016), while maintaining a healthy body weight (WCRF/AICR, 2018). Indeed, many Food-Based Dietary Guidelines in Europe recommend an increased consumption of legumes (EC, 2023). Despite this, global legume consumption remains low. In 2019, diets low in legumes, defined as “average daily consumption (in grams per day) of less than of 90–100 g of legumes and pulses, including fresh, frozen, cooked, canned, or dried legumes” (IHME, 2019a), were the fourth-leading dietary risk factor for attributable disability-adjusted-life-years (DALYs; one DALY represents the loss of the equivalent of one year of full health), accounting for 24.3 million DALYs and 1.12 million avoidable deaths from all causes (IHME, 2019b). This highlights the urgency of promoting legume consumption to improve public health.

### 1.2 Barriers to the consumption of new, healthier and more sustainable legume-based foods

Taste preference and dietary habits are shaped early in life and can become established and sustained (Larson et al., 2008, Mikkilä et al., 2005). Adolescence, spanning ages 10–19 according to the World Health Organization (WHO), represents a pivotal phase for establishing lifelong healthy eating habits (WHO, 2022a). It marks a significant transition from relying on caretakers to assuming greater responsibility for food choices, procurement, and consumption. Thus, adolescence offers a critical opportunity to foster healthy and sustainable eating habits (Larson et al., 2008, Neufeld et al., 2022). Studies have shown that although adolescents know what constitutes healthy food choices and their importance for overall well-being, translating this knowledge into practice remains a challenge (Neufeld et al., 2022). In fact, adolescents’ food choices are influenced by different factors under the social context in which they live, including family, community, peers and school, as well as by their quest for independence, beliefs, convenience, taste, texture and liking (Bronfenbrenner and Morris, 2006, Demory-Luce and Motil, 2022, Dinnella et al., 2016, Morgan et al., 2016). In particular, taste, texture and liking play a strong role in adolescents’ food choice, as they can act both as facilitators and as barriers to healthy and sustainable eating (Kebbe et al., 2017). Innate preferences shape adolescents’ appreciation for vegetables (Dinnella et al., 2016; Poelman et al., 2017). For instance, adolescents tend to prefer vegetables characterized by innately liked tastes, such as sweet and umami, as well as more delicate flavours and brighter colours, while rejecting those with innately disliked sensations such as bitter and sour tastes (Bawajeeh et al., 2020, Cox et al., 2012, Dinnella et al., 2016, Hartwell et al., 2020). Additionally, objectionable flavours and unappealing textures including hard/hard-skinned, slimy and granular textures are often avoided (Appleton et al., 2019, Dinnella et al., 2016, Hartwell et al., 2020, Krølner et al., 2011). The sensory properties of legumes have been increasingly investigated due to their importance as source of plant-based (PB) proteins (Tso et al., 2020). Concerns about lack of taste, mush texture and dull colour are reported as the main reasons for their low sensory appeal among children and young adults (Poelman et al., 2017; Spencer et al., 2018).

Food neophobia (FN), which refers to the reluctance to eat or avoidance of novel foods (Pliner & Hobden, 1992), acts as a barrier to food appreciation and consumption, particularly of foods with bitter taste, poor texture and appearance and objectionable or stronger flavours (De Toffoli et al., 2019, Dovey et al., 2008, Laureati et al., 2015), characteristics often present in many vegetables and legumes (Dovey et al., 2008). Lower FN levels have been associated with higher vegetable liking and consumption (Guzek et al., 2017, Laureati et al., 2018, Mielby et al., 2012), as well as increased diet variety and adherence to diets such as the Mediterranean diet (Karaağaç & Bellikci-Koyu, 2023). FN typically peaks during early childhood, gradually decreasing until it stabilizes during adolescence or early adulthood (Dovey et al., 2008, Hazley et al., 2022). Therefore, interventions aimed at increasing adolescents' consumption of PB foods should carefully consider FN’s influence on food choices.

Research on children's preferences for vegetables and legumes indicates notable differences by gender, with girls showing a higher preference for vegetables compared to boys (Cooke & Wardle, 2005). Furthermore, several studies showed that socioeconomic status (SES) affects food choices (Darmon and Drewnowski, 2008, Hough and Contarini, 2023, Hough and Sosa, 2015, Inglis et al., 2008). Low-income populations face significant barriers to accessing nutritionally adequate foods. High-energy, low-cost foods contribute to overnutrition but lack essential nutrients, leading to simultaneous overnutrition and micronutrient deficiencies (Hough & Sosa, 2015). Furthermore, research has shown that the cost is a significant barrier for a shift towards healthier and more sustainable diets among low-income consumers. In fact, sustainable food options like organic and PB alternatives are generally more expensive than conventional alternatives, making them less accessible to low-income consumers (Hough & Contarini, 2023). Research consistently shows that adolescents from lower SES consume fewer healthier foods, such as vegetables, and more unhealthy foods, such as fast foods and soft drinks, compared to their more affluent peers (Bel-Serrat et al., 2023; Delbosq et al., 2022, Skårdal et al., 2014). Despite this, adolescents from lower SES remain an under-investigated group (Corrington et al., 2020).

These findings underscore the pressing need for more targeted efforts to increase adolescents’ legume consumption, considering sensory factors such as taste, texture, appearance, liking, and novelty, and ensuring inclusion of adolescents from lower SES. Interventions promoting legumes, renowned for their health benefits and affordability, could enhance the quality of their diet. However, school interventions targeting adolescents and promoting legumes are limited, particularly those focusing on enhancing the sensory characteristics of this food group (Kokkorou et al., 2024).

### 1.3 The concept of co-creation in developing tailor-made dishes

An effective strategy to overcome these barriers to PB and LB food consumption is to engage adolescents to a high degree in the innovation process. This ensures that the new foods are tailor-made to their preferences, particularly in terms of sensory properties and their impact on emotional responses. In fact, given the high level of adolescents’ autonomy and agency compared to children —referring to their ability to make independent decisions and act on them— there is great potential for them to be active drivers of change encouraging healthier and more sustainable eating (Neufeld et al., 2022). The adoption of co-creation methods offers a promising avenue to integrate adolescents’ perspectives into the development of innovative LB foods with appealing sensory properties. Co-creation is a valuable approach/methodology that indicates a shift from the producer/organisation in defining the product to a more participatory process in which consumers, and all stakeholders more broadly, actively contribute to idea generation (Baldwin and von Hippel, 2010, De Koning et al., 2016). Indeed, co-creation is one of the most used forms of citizen science (Haklay et al., 2020, Reynolds et al., 2021). Its participatory approach enables the development of products and services that are better appreciated by the end-users while reinforcing consumer engagement in the co-creation process. Co-creation fosters a sense of ownership and involvement among participants, as they are directly involved in the design and development of solutions. Therefore, well-designed co-creation activities could not only develop more accepted healthier and sustainable foods, but also empower adolescents to explore their way into healthy and pleasurable eating.

From a methodological perspective, co-creation is not identified by a univocal approach and there is still limited methodological research with children and adolescents on co-creation activities aiming at generating new foods (Driessen-Willems et al., 2021, Galler et al., 2022). Moreover, existing studies employing co-creation with children and adolescents often overlook SES considerations (Driessen-Willems et al., 2021) or acknowledge potential underrepresentation of adolescents from different backgrounds such as lower SES and immigration status. Input from these population groups could significantly enhance the acceptability of the final products from a wider variety of subjects.

Various methods have been applied for co-creation activities and idea generation, encompassing focus group discussions (Bruseberg & McDonagh-Philp, 2002), free-association-tasks (Koll et al., 2010), the Substitute-Combine-Adapt-Modify-Eliminate-Reverse (SCAMPER) technique (Eberle, 1972, Serrat, 2017), and the Jobs-To-Be-Done (JTBD) framework (Ulwick, 2017). The focus group discussions are recognised as effective strategies for brainstorming (Banović et al., 2016, Boonpracha, 2023, Galler et al., 2022), while free-association-tasks aim to identify consumers’ perceptions and attitudes towards specific products or brands (Koll et al., 2010). SCAMPER represents a strategy and methodology that employs brainstorming and mind mapping techniques. It aids in diverging from conventional logic, fostering a broad spectrum of innovative ideas and perspectives when addressing problems requiring creative solutions. It has been successfully applied to idea creation and product design (Boonpracha, 2023). The JTBD framework is based on the theory of (disruptive) innovation, that states that people buy products and services to fulfil specific “jobs”, which may be functional (e.g., to be fed), emotional (e.g., to feel relaxed) or social in nature (e.g., to make new acquaintances) (Christensen & Raynor, 2003). Identifying the JTBD has been indicated as a preliminary step necessary for successful product innovation (Ulwick, 2017). While a variety of methods and approaches is available for co-creation, their application to food co-creation is limited (Banović et al., 2016, Galler et al., 2022).

Emotions, characterised by valence (positive or negative) and arousal (degree of physiological activation) are very important in new product acceptance (Spinelli, 2021). Product consumption elicits emotions (a product can make us feel happy, relaxed etc.) and new products often evoke surprise upon first experience. After a while, surprise decreases and may evolve in different emotions that can be positive (e.g., relaxing, cheerful) or even negative (e.g., boredom) (Köster & Mojet, 2007). For these reasons, considering emotions in new product development is crucial for the success of the product. Building on the promising effect of co-creation in developing tailor-made dishes and overcoming sensory barriers, as well as the recognized benefits on legumes for human and environmental health, this study aimed to develop innovative, sustainable and healthy LB dishes for school canteens using a co-creation approach with adolescents (including those from low SES). In particular, the study aims to address gaps in current research by integrating liking responses with measures of emotional responses to products in the product design and development process. Furthermore, the study aims to develop solutions that are accepted by all adolescents, including those higher in FN. This was done by considering individual differences in the acceptability of new foods related to FN within the innovation and co-creation process.

## 2 Materials and methods

### 2.1 Participants and setting

The study was conducted at Istituto Professionale Buontalenti, a technical secondary school of Hospitality and Enogastronomy, where future chefs for institutional food services, canteens and restaurants are trained. The school does not have a traditional canteen but several kitchens and areas for serving the food prepared as an integral part of the school offer. The new dishes were co-created with adolescents using the evoked context technique to develop innovative LB dishes for school canteens (i.e. adolescents were asked to imagine being a chef in a school canteen (Hein et al., 2012) and to evaluate them (i.e. adolescents were asked to imagine being in a school canteen while tasting the samples). Additionally, chefs/teachers of the culinary school participated in the co-creation process to optimize the quality and acceptance of the final recipes, ensuring they are feasible for preparation in school canteens.

The school is located in the south-west suburb of Florence, in a populated district with the lowest per-capita income of the town (Comune di Firenze, 2023). With around 1100 students annually, the school offers an interesting setting to study food innovation in an inclusive and more representative context, where also students from lower SES are considered. Around 25 % of the students have immigrant backgrounds (primarily from Eastern Europe, Asia, and Africa). This percentage is notably higher than the regional average of 14.2 % (Ministero dell’Istruzione, 2020) for students with immigrant backgrounds (2nd–3rd generation), indicating a greater diversity. Furthermore, around 30 % of students at the school present special educational needs, including language barriers, specific learning disorders and physical disabilities. Moreover, as a technical secondary school of Hospitality and Enogastronomy, it allows for collaborations with professional chefs who are also teachers. The canteen functions uniquely, serving as a practical learning space for students studying hospitality. Here, students learn to cook and serve meals, which are provided to other students as part of their educational experience.

Data were collected between October 2022 and January 2023. In total 265 adolescents 14–17 years old participated in the different steps of the study. Adolescents were recruited among students attending the first or second grade of the technical secondary school. Eleven classes were recruited in total; even if not all the students participated in the study for various reasons, such as lack of parental consent, personal non-consent, or absence on the day of the tests, the average participation rate per class was 98 %. An additional inclusion criterion for the students evaluating/tasting the dishes was having no food-related allergies to the ingredients. Demographic characteristics of the six panels participating in each co-creation step are reported in Table 1. As our primary objective was to amplify the voices of low SES adolescents in the co-creation journey rather than to compare ideas for new products across subjects differing in SES, detailed socio-demographic information on the panels was not collected. Written informed consent was obtained from all subjects and parents in line with the General Data Protection Regulation (GDPR) 2016/679. Confidentiality and anonymity were maintained through secure data storage and pseudonymisation of participant information. Appropriate health and safety considerations, together with a risk assessment protocol, were carried out by the school personnel prior to the commencement of the research. This study was conducted according to the principles established in the Declaration of Helsinki (World Medical Association, 2013) and approved by the Board of the School “Istituto Professionale Buontalenti” and the Department of Agriculture, Food, Environmental and Forestry (DAGRI), University of Florence.

**Table 1:** Co-creation steps, demographic characteristics of the panels who participated in each co-creation step. SD = Standard Deviation; FAT = Free Association Tasks; WTT = Willingness To Try; RGM = Repertory Grid Method; UniFi = University of Florence; n/a = not applicable.

Co-creation & dish development steps	Aim	Methods	No. of concepts/dishes	Panels	n	Age (mean, SD)	Men (%)
Step 1. Identification of opportunities and concept development	Concept identification of new LB dishes	Focus Groups, JTBD, FAT, SCAMPER	11	Adolescents (A1)	19	15.3 (0.73)	63
		Focus Groups SCAMPER	5	Chefs	4	n/a	100

Step 2. Concept refinement	To refine the concepts of new LB dishes	SCAMPER	28	Adolescents (A1)	19	15.3 (0.73)	63

Step 3. Concept validation	To test concept attractiveness	WTT	28	Adolescents (A2)	91	14.9 (0.73)	59

Step 4. Concept selection & recipe development	To select the dishes to prepare	Nutritional & sustainability assessment	12	UniFi researchers	3	n/a	33
		Assessment of cost, feasibility & preparation/ serving time.	12	Chefs	4	n/a	100

Step 5. Generation of dish specific sensory and emotional vocabulary	Development of the sensory & emotional questionnaire	Modified RGM (EmoSemio)	6	Adolescents (A3)	17	14.9 (0.83)	65

Step 6. Prototype validation	To select the dishes with the best affective impact	Liking, Sensory & Emotional Profile	5	Adolescents (A4)	138	14.7 (0.76)	66

### 2.2 Procedure

**Fig. 1:**
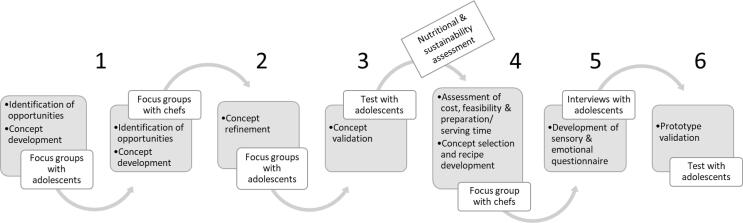
The co-creation process: from the identification of opportunities to prototype validation.

A multimethod setup with six steps was originally designed for the co-creation process 1) Identification of opportunities and concept development, 2) Concept refinement 3) Concept validation, 4) Concept selection & recipe development, 5) Generation of dish specific sensory and emotional vocabulary, and 6) Prototype validation (Fig. 1). This six-step process is grounded in established co-creation methodologies, based on the frameworks provided by Sanders and Stappers (2008) and Steen et al. (2011), which emphasize iterative ideation, prototyping, and evaluation phases. This approach ensures active participant involvement throughout the development process, enhancing the relevance and acceptance of the LB dishes. This section outlines each step, the data collection methods used, and the subsequent data analysis procedures.

For the qualitative steps (step 1 & 2 (concept identification/refinement) and step 5 (the development of the dish-specific questionnaire)), small panels were preferred for ensuring successful sessions (classes with 19 and 17 adolescents, respectively). For the quantitative steps (concept (step 3) and dish (step 6) validation), larger panels were recruited in accordance with published literature suggesting a sample size near 100 for stable averages and significant difference identification in product testing (Gacula and Rutenbeck, 2006, Hough et al., 2006, Moskowitz, 1997): 91 and 138 adolescents were retained for steps 3 and 6, respectively. These sample sizes ensure robust tests, with a near 100 % chance of correctly rejecting the null hypothesis for the different effects considered in the ANOVA models.

#### 2.2.1 Step 1: Identification of opportunities and concept development with adolescents and chefs

This step aimed to identify opportunities to improve the school canteen experience and to develop new legume-based dishes. Furthermore, it aimed to define concepts for new LB dishes, providing initial ideas or blueprints for a new food product. These concepts included a short product description highlighting key features that differentiate them from existing products.

In this step, adolescents engaged in brainstorming sessions to enhance school canteens and develop ideas of new LB main dishes. This process involved a combination of methods, including focus groups (Banović et al., 2016, Galler et al., 2022), Jobs-To-Be-Done (Ulwick, 2017), free-association-tasks (Koll et al., 2010) and SCAMPER (Boonpracha, 2023, Serrat, 2017). Nineteen adolescents (panel A1) were divided into three focus groups (n ≈ 6 each, 1-hour duration), each led by a trained moderator and an assistant. A multimethod setup with three stages was designed to allow the gradual exploration of the topic. Adolescents were tasked with envisioning themselves as head chef of a school canteen, responsible for selecting the new menu (using the immersive approach of the evoked context, Hein et al., 2012). Initially, the Jobs-To-Be-Done framework was employed, prompting students to identify shortcomings in current school canteen offerings and articulate their ideal expectations for school meals and which “jobs” they are hired for (e.g., as a social moment, to be fed…). Subsequently, the free-association-tasks technique was used. Focus groups were presented with nine cards, each featuring a different commonly consumed legume in Italy — brown lentils, red lentils, soybeans, chickpeas, fava beans, green peas, soybean sprouts, borlotti (brown) beans and cannellini (white) beans (these data are not discussed here). Participants were then asked to provide three words they associated with each legume (e.g., winter, sadness). This step aimed to gauge their knowledge and attitudes toward legumes, with results represented as word-clouds (Supplementary materials 1). Finally, students engaged in concept development for new LB dishes. They were invited to imagine themselves as chefs in a school canteen, tasked with developing new legume-based dishes to replace meat dishes on the menu one day per week (evoked context). Each group was asked to generate a minimum of three innovative dish concepts using at least one of the legumes presented on the cards for each dish. They were also required to exclude meat, pasta, or rice from the dishes. Meat was excluded because the goal was to create alternative protein-based dishes, while pasta and rice were excluded because they are typically part of the first course in Italy, and the new dish was conceived as a second course. During the focus group, students were guided through a series of questions designed to stimulate their creativity and encourage them to explore various aspects of dish creation (e.g. combination/substitution of ingredients) through the adaptation of the SCAMPER technique (Table 2). The last question aimed to develop variants of the same concepts, such as changing an ingredient or the method of preparation.

**Table 2:** Example of the adapted version of the SCAMPER technique that was applied with the adolescents and chefs to develop new concepts of LB main dishes for school canteens (translated from Italian).

**Questions**	**Answers (examples)**
Instead of meat you can use one or more legumes of your choice among those on the cards. Which one?	e.g., chickpeas; beans
What other ingredients would you combine them with?	e.g., cherry tomatoes, basil
Why did you choose these ingredients?	e.g., to make a cheerful colour contrast
What dish will you create? (i.e. do you think of a dish that is similar to the one you would like to create and that you have taken as a model?)	e.g., a kind of salad
What do you think are the strong points of the dish?	e.g., freshness
What could you change in this recipe if you were to think of a variant? (e.g., you could change an ingredient, or the chickpeas could be puréed, etc.)	e.g., chickpeas can be removed and we may add corn to make it more colourful

Following the students’ co-creation activities, chefs participated in a focus group discussion. Chefs’ participation aimed to increase the variety of the identified concepts, as well as the ingredients and cooking methods proposed. Initially, they shared perspectives on the JTBD for meals in school canteens. Subsequently, they discussed insights gleaned from students on this same topic. Furthermore, they were shown the word-clouds generated from students on this topic. Using the SCAMPER technique, chefs were then guided to develop concepts for new LB dishes, following a similar approach to the one used with students, starting by selecting a legume as main ingredient. Chefs were asked to consider the word-clouds when proposing dish concepts, with each of them selecting at least one legume. All sessions were recorded, and data of the dish concepts were collected using structural grids containing predefined categories and questions (e.g., main ingredient, additional ingredients, strong characteristics, possible variations). This ensured that the relevant information was captured uniformly and comprehensively, facilitating a structured approach to data analysis.

#### 2.2.2 Step 2: Concept refinement with adolescents

Building on the initial concepts, this step aimed to refine and improve the ideas based on further inputs from adolescents employing the SCAMPER technique. The same groups of students (panel A1) involved in the initial focus group sessions reviewed the concepts developed by the other groups and by the chefs. They were tasked with adjusting these concepts, focusing on the last two questions of the SCAMPER framework (Table 2). Potential changes encompassed ingredient substitutions or additions, alterations in cooking methods, or ingredient elimination.

#### 2.2.3 Step 3: Concept validation with adolescents

This step aimed to validate the refined concepts with a larger group of adolescents, by assessing their willingness to try (WTT) each dish (presented as a concept). All concepts generated from the focus groups with adolescents and chefs underwent analysis and were consolidated in cases of duplication. Short descriptions were crafted for each concept (Table 3). An online questionnaire, designed to measure students’ WTT the concepts, was developed. Students (panel A2) completed this questionnaire using tablets and computers (Compusense, 2023) while situated in their classroom. The questionnaire consisted of three sections: 1. Demographics (age, gender, school class), 2. The Italian Child Food Neophobia Scale (ICFNS) (Laureati et al., 2015) was used to measure FN consisting of eight statements (4 neophilics and 4 neophobics) evaluated on a 5-point-categorical scale (1: very false, 5: very true), 3. WTT the dishes (presented using concept description) was assessed using a 9-point-categorical scale (1: I really don’t want to taste it – 9: I would very much like to taste it). The concepts’ descriptions appeared in a balanced order to avoid presentation order effect.

**Table 3:** Concepts of the co-created dishes (translated from Italian), their relative characteristics (main legume ingredient, type of dish, temperature of serving and main sensory characteristics), Willingness-To-Try (WTT) mean score and standard deviation (SD). Letters indicate a significant difference (p < 0.050) as determined by Tukey (HSD).

**Code**	**Concept of dishes**	**Legume used**	**Type of dish**	**Serving temperature**	**Main sensory characteristics**	**WTT**	
						**Mean**	**SD**
7	A plate of fresh and colourful poké, with soybean sprouts, peas, crunchy salad, corn, chopped tomatoes, avocado, sea bass and soy sauce	Soybean sprouts, peas	Poké, fresh, ethnic	Cold	Crunchy, colourful	6.58a	2.24
**19**	Lentil “arancino” stuffed with chopped lentil cream with crunchy lentil breading and with a heart of tomato sauce inside	Lentils	Ball made of lentils	Warm	Crunchy, creamy	6.36ab	2.16
24	Grilled polenta sandwich with lemon chickpea cream (hummus) inside, stringy mozzarella and chopped tomatoes	Chickpeas	Sandwich	Warm	Creamy, contrasting flavours	6.11abc	2.23
18	Lentil “arancino” stuffed with chopped lentil cream with crunchy lentil breading and with a cube of salmon inside	Lentils	Ball made of lentils	Warm	Crunchy, creamy	6.02abcd	2.38
2	Soy noodles soybean sprouts, grilled peppers, zucchini, sautéed julienne carrots, strips of omelette, oil, salt, pepper, oregano, parsley, soy sauce	Soybean sprouts, soybeans	Pasta, ethnic	Warm	Colourful, soft	5.90abcde	2.44
1	Soy spaghetti with soybean sprouts, grilled peppers and zucchini and cherry tomatoes sautéed with oil, salt, pepper, oregano, parsley, soy sauce	Soybean sprouts, soybeans	Pasta, ethnic	Warm	Colourful, soft	5.78abcdef	2.48
**6**	A fresh and colourful dish, with crunchy salad, corn, soybean sprouts, peas, avocado, pecorino cheese flakes, chopped tomatoes and soy sauce	Soybean sprouts, peas	Fresh, salad	Cold	Colourful, crunchy	5.75abcdef	2.41
**27**	Lentil-based burger with avocado sauce, tomatoes	Lentils	Burger	Warm	Colourful, creamy, contrasting flavours	5.75abcdef	2.22
5	A fresh and colourful dish, with crunchy salad, corn, soybean sprouts, peas and tomatoes cut into small pieces, crispy parmesan wafer	Soybean sprouts, peas	Fresh, salad	Cold	Colourful, crunchy	5.71abcdef	2.29
20	“Crostone” with pea cream with baby squid, parsley, and lemon	Peas	Sandwich	Warm	Crunchy, contrasting flavours	5.56abcdef	2.61
8	A fresh and colourful dish, with crunchy salad, fresh broad beans (pods), diced tomato, pecorino flakes and crispy bread	Soybean sprouts, peas	Fresh, salad	Cold	Colourful, crunchy	5.51abcdefg	2.28
25	Warm “crostone” with cannellini beans, chopped vegetables as decoration served with an oil flavoured with herbs that each can be added as desired	Cannellini beans	Sandwich	Warm	Colourful, crunchy	5.51abcdefg	2.00
16	Tomatoes cut in half stuffed with pea cream, eggplant, and mozzarella au gratin in the oven	Peas	Stuffed tomato	Warm	Soft, creamy	5.45bcdefg	2.68
9	Savory pie with zucchini, eggplant, peas, peppers, potatoes, egg, and cheese.	Peas	Pie	Warm	Soft	5.44bcdefg	2.71
13	Tomatoes cut in half filled with chickpea cream and baked au gratin in the over with grilled vegetables for decoration	Chickpeas	Stuffed tomato	Warm	Creamy, soft	5.41bcdefg	2.37
10	Pea and soy salad with soy sauce, diced tomatoes, carrots and cucumbers, cheese flakes, onion chips or caramelized onion	Peas, soybeans	Fresh, salad	Cold	Crunchy, contrasting flavours	5.33bcdefgh	2.53
15	Halved tomatoes stuffed with chickpea cream and lentils with grilled peppers to decorate and yogurt sauce	Chickpeas, lentils	Stuffed tomato	Warm	Creamy, contrasting flavours	5.30bcdefgh	2.53
17	Lentil arancino stuffed with chopped lentil cream with crunchy lentil breading and with a pumpkin and carrot heart inside.	Lentils	Ball of lentils	Warm	Crunchy, creamy	5.27bcdefgh	2.59
4	A fresh and colourful dish, with soybean sprouts, fresh broad beans (pods), crunchy lettuce, corn and chopped tomatoes	Soybean sprouts, broad beans	Fresh, salad	Cold	Crunchy, colourful	5.13cdefgh	2.39
14	Pepper cut in half au gratin in the oven stuffed with cream of lentils, eggs, herbs and tomatoes	Lentils	Stuffed pepper	Warm	Creamy	5.13cdefgh	2.57
11	Pea, red bean and soy salad with soy sauce, diced tomatoes, cheese flakes, onion chips or caramelized onion	Peas, red beans soybeans	Fresh, salad	Cold	Crunchy, contrasting flavours	5.12cdefgh	2.51
22	Cold rice with peas, corn, olives, parsley, lemon, and turmeric	Peas	Fresh, rice salad	Cold	Contrasting flavours	5.12cdefgh	2.52
3	Russian salad with beans, peas, carrots, capers, and yogurt sauce	Beans, peas	Fresh, salad, ethnic	Cold	Creamy	5.01cdefgh	2.53
23	Mille-feuille with chickpeas: crunchy layers with lemon chickpea cream (hummus) inside	Chickpeas	Sandwich	Warm	Crunchy, creamy, contrasting flavours	4.96defgh	2.31
26	Soup with cannellini beans and chopped vegetables as decoration served with an oil flavoured with herbs that each can be added as desired	Cannellini beans	Soup	Warm	Liquid, soft, colourful	4.80efgh	2.39
28	Soup of lentils, onions, garlic, oregano, and tomato	Lentils	Soup	Warm	Liquid, soft	4.75fgh	2.70
12	Soup with soybean sprouts, hard-boiled egg, ginger, miso, bamboo	Soybean sprouts	Soup, Ethnic	Warm	Liquid, soft	4.45gh	2.49
21	“Crostone” with pea cream with anchovies, parsley, and lemon	Peas	Sandwich	Warm	Crunchy, creamy, contrasting flavours	4.27h	2.43

#### 2.2.4 Step 4: Concept selection & recipe development with chefs

This step focused on selecting the most promising concepts based on WTT scores of more neophobic and neophilic adolescents and developing recipes that meet certain criteria (nutritional, sustainability, suitability for school canteens).

Following the analysis of the WTT scores, 12 concepts were selected for further evaluation, prioritizing concepts that best matched the following criteria: highest mean WTT across all subjects, mean WTT score above the group average for high-food neophobia (HFN) individuals, and highest mean WTT scores for low-food neophobia (LFN) individuals. The selected concepts were then qualitatively assessed for environmental sustainability and adherence to Mediterranean diet principles by the UniFi research team using binary variables (YES/NO question). Sustainability was evaluated based on whether the ingredients in the concept descriptions had high environmental impact, according to estimates provided by Clark et al. (2022). Adherence to the Mediterranean diet was determined by assessing whether the estimated macronutrient ratios of the concepts aligned with Mediterranean diet’s principles (carbohydrates: 55–60 % of the total energy, proteins: 15 %, fat: 30–35 %) as recommended by Davis et al. (2015) and Ge et al. (2020). Additionally, the accepted concepts were evaluated by the chefs using binary variables (YES/NO question) to assess their cost, feasibility and preparation/serving time suitability for school canteen use (Table 4). Given that the final dishes were intended for school canteens, it was essential for them to be affordable, feasible and quick to prepare/serve. During this step, chefs provided suggestions to enhance some of the recipes based on the five aforementioned criteria. Subsequently, chefs developed recipes for the six concepts that best met these criteria, which were then evaluated by the UniFi team. During the preparation process, minor adjustments were made to enhance the texture, taste, or address any preparation constraints of the recipes. The nutritional composition of each recipe was calculated to define an appropriate portion size, following the national guidelines for school canteens (Ministero della Salute, 2010). The six dishes selected for the next step (Step 5) included tomato arancino (with lentils), pumpkin arancino (with lentils), lentil-based burger, polenta sandwich (with chickpeas), stuffed tomato (with chickpeas) and a corn and pea soybean sprout salad dish.

**Table 4:** Concepts’ assessment from a nutritional (adherence to Mediterranean Diet) and environmental sustainability point of view and from the chefs against the criteria of cost, preparation/servings time and feasibility. 'YES' responses (green shaded), 'NO' responses (red shaded). In bold are the six concepts selected for preparation after revision.

*This concept was selected with the requirement to substitute salmon with pumpkin.

#### 2.2.5 Step 5: Generation of dish specific sensory and emotional vocabulary

This step aimed at identifying sensory and emotional descriptors for the selected dishes to develop a product-specific questionnaire to be used for prototype evaluation. The questionnaire was developed based on one-on-one interviews with 17 adolescents (panel A3) conducted with a modified version of the Repertory Grid Method, EmoSemio (Spinelli and Jaeger, 2019, Spinelli et al., 2014), to identify the main sensory and emotional descriptors of the six developed dishes. Adolescents were asked to first taste and rank the dishes according to their preference. Then the dishes were divided in two triads. All six dishes were presented in small samples to the adolescents in a balanced presentation order. The interviewee was asked to taste the six dishes and rank them based on his/her preference. Subsequently, the dishes were divided into two triads (1st–3rd and 4th–6th in the ranking) according to the respondent’ ranking. Starting with the first triad (the most preferred dishes), the interviewee was asked to report how each dish makes him/her feel in comparison to the other two and then the question was repeated for sensory differences among dishes (“How does this dish that you indicated as first in the ranking make you feel compared to the other two?”; “In which way is it different from the other two?”). The same procedure was repeated for all the dishes in the triad, and then for the second triad. A semiotic analysis was conducted on the interviews to identify the semantic categories underlying adolescents' perception of the dishes. Based on this, a questionnaire was developed to measure sensory and emotional responses to the developed dishes. The semantic categories were then structured into sentences to make the meaning clearer for the participants (Table 5, Table 6). We refer to Spinelli and Monteleone (2023) for a detailed description of the protocol. Participants were instructed to rinse their mouth using drinking water between two samples.

**Table 5:** Dish specific emotional questionnaire developed using the EmoSemio procedure.

**Emotions**	
**Full sentence**	**Short version**
It makes me cheerful	Cheerful
It excites me	Enthusiastic
It gives me a sense of pleasure	Pleasure
It makes me feel satisfied	Satisfied
It makes me curious	Curious
It inspires me	Inspiring
It’s mouth watering	Mouth watering
It surprises me	Surprised
It makes me feel full of energy	Energetic
It makes me sad	Sad
It makes me feel depressed	Depressed
It disappoints me	Disappointed
It disgusts me	Disgusted
It’s Indifferent to me	Indifferent
It makes me feel nostalgic	Nostalgic

**Table 6:** Dish specific sensory questionnaire developed using the EmoSemio procedure.

**Sensory descriptors**	
**Full sentence**	**Short version**
It’s sweet	Sweet
It’s bitter	Bitter
It has an acid/sour taste	Sour
It's salty	Salty
It's tasty	Tasty
It has a delicate flavour	Mild flavour
It has a strong flavour	Strong flavour (F)
It has a spicy taste	Spicy flavour (F)
It has a fresh taste	Fresh flavour (F)
There is a contrast of flavours	Contrasting flavours (F)
It melts in your mouth	Melting
It's crunchy	Crunchy
It's soft	Soft
It has a compact texture	Firm
It's dry	Dry
It's grainy	Grainy
It's hard	Hard
It's creamy	Creamy
It's hard to chew	Difficult to chew
It crumbles	Crumbling
It's colourful	Colourful
It's complex	Complex

#### 2.2.6 Step 6: Prototype validation

The final step aimed at testing the developed prototypes with a large group of adolescents to evaluate sensory and affective responses using the product-specific questionnaire developed in the previous step. Prototype validation occurred over two days in the school’s dining room, where students (panel A4) (n = 138, mean age = 14.7 (0.76) were seated at round tables, allowing for individual evaluation (8 students per table). Five prototypes were considered for the study. One (the salad) was excluded because it was not suited as a main dish for its nutritional composition. The test was conducted using tablets that gave access to the online questionnaire designed to measure sensory and affective responses to selected dishes. Initially, students were asked to provide their age and gender. Then, they were served a small portion of the five dishes presented in a balanced order, each identified by a 3-digit code. For each sample, subjects rated their liking on a Labeled Affective Magnitude (LAM) (Cardello & Schutz, 2004), followed by emotions and the sensory properties rated on a 7-point scale (1: not at all – 7: very very much) (Table 5, Table 6). Between samples, students were instructed to rinse their mouths with drinking water. Finally, students filled out the FN questionnaire as described above. Before commencing the test, students received instructions on using the different scales and were informed about the study’s aim (developing new recipes for school canteens). They were also asked to imagine they were tasting these dishes in a school canteen (evoked context). Assistants were present at each table to guide and assist students as needed.

### 2.3 Data analysis

#### 2.3.1 Steps 1 & 2: Concept development and refinement

Qualitative data were gathered from focus groups and brainstorming sessions involving adolescents and chefs. The information was analyzed to identify common themes and insights, which were then used to develop dish concepts.

Each focus group was recorded. The information collected in the focus groups was qualitatively analysed to identify the sensory, social, functional and emotional JTBD of food served in school canteens.

Three interviewers independently performed a semiotic analysis of the focus group data, which was then discussed collectively to define the concepts. Word-clouds were created to visualize the key words associated with each legume.

#### 2.3.2 Step 3: Concept validation

Quantitative data were collected using an online questionnaire designed to measure students’ willingness to try each of the dishes (presented as concepts) and FN.

Cronbach’s alpha (0.64 for step 3 and 0.8 for step 6) was calculated for reliability, after reversing the items in line with Laureati et al. (2015). The individual FN scores were computed as the sum of ratings given to eight statements (using a 5-point-categorical scale), after the neophilic items had been reversed. The scores range from 8 to 40, with higher scores reflecting higher FN. Adolescents were split around the median (step 3: FN median = 20, step 6: FN median = 21) in low and high neophobic subjects (LFN and HFN respectively).

A Two-way ANOVA model was computed to test “concept of dish” and “subject” effects on WTT. Two-way ANOVA models were also used to test the effect of “concept of dish” and “gender” and their interaction, or “concept of dish” and “FN level”, and their interaction on WTT. Tukey’s HSD test (honestly significant difference) was used as a post hoc test when a significant difference was detected in the ANOVA (p < 0.050). Cohen's f statistic was used as a measure of effect size in the ANOVA models.

#### 2.3.3 Step 4: Nutritional and sustainability assessment and recipe selection

Selected concepts from the validation step were evaluated using Yes/No responses for criteria such as sustainability, adherence to the Mediterranean diet, cost, feasibility, and preparation time. Responses were counted and discussed by a multidisciplinary group. Recipes were developed and adjusted based on these criteria and qualitative feedback from chefs.

#### 2.3.4 Step 5: Generation of dish specific sensory and emotional vocabulary

Qualitative data were gathered from one-on-one interviews using the modified Repertory Grid Method, to identify key descriptors (Spinelli et al., 2014). A semiotic analysis was conducted on the interviews to identify the emotional and sensory dimensions relevant to adolescents when experiencing the new dishes (Spinelli and Jaeger, 2019, Spinelli and Monteleone, 2023, Spinelli et al., 2014). These were subsequently translated into short sentences by the UniFi research team (emotions Table 5 and sensory properties Table 6), intended for use in the questionnaire in the next step.

#### 2.3.5 Step 6: Liking, sensory and emotional responses to tested dishes

The prototypes were served to students in a school dining room, and their responses were collected using a questionnaire measuring FN, liking, emotions, and sensory properties of the dishes.

A Two-way ANOVA model was computed to test “dish” and “subject” (within-subjects factors) effects on liking. “FN level” and “gender” effects (as between-subjects factors) on liking scores were tested in two independent Two-way ANOVA models along with “dish” effect and the relative two way interaction.

Sensory properties were initially submitted to a Two-way ANOVA model with “dish” and “subject” as within-subjects factors. Only attributes with a significant dish effect (p < 0.050) were selected to map sensory differences among products by means of a Principal Component Analysis (PCA). Furthermore, to study individual differences in liking an Internal Preference Map (IPM), i.e., a Principal Component Regression (PCR), was computed (Unscrambler version 10.1, Camo), with individual liking scores as X matrix (dish x subjects data) and significant sensory attribute as Y matrix (dish x mean scores of significant sensory attributes). To improve visual interpretation, samples were included as dummy variables (down-weighted in the data matrix).

Exploring individual differences in liking and finding consumer segments are among the reasons to apply preference mapping. Looking at the correlation loadings of the IPM, three components were retained. In these cases when the number of relevant principal components is larger than 2, segmentation based on visual inspection of IMP plots can be supported by either a hierarchical or a criterion-based clustering method (Næs et al., 2018, Pierguidi et al., 2020). Thus, a hierarchical clustering (AHC) on subject correlation loadings in the first 3 PCs of the IPM was used to identify subject clusters differing in liking patterns. The nature and integrity of the clusters were checked following the recommendations of Næs et al., 2018, Endrizzi et al., 2014 and further confirmed by an ANOVA model on liking, with “cluster” as between-subjects variable and “dish” as within-subjects variable.

Tukey’s HSD test was used as a post-hoc test when a significant difference was detected in the ANOVA (p < 0.050). Cohen's f statistic was used as a measure of effect size in the ANOVA models.

Segments were compared for gender using a χ^2^ test, also testing significance by cell using Fisher’s exact test (p > 0.050). Cluster effect on FN score was tested by means of a One-way ANOVA model.

To investigate the emotional responses associated with the dishes, we looked at the Consensus Space on emotions configuration of cluster 1 and cluster 2 after performing a Generalized Procrustes Analysis (GPA). GPA is a multivariate exploratory technique (multiblock method) that involves transformations (i.e., translation, rotation, reflection, isotropic rescaling) of individual data matrices to provide optimal comparability between blocks of data, e.g., between subjects or clusters. An iterative process refines the alignment to achieve the best possible fit, minimizing the Procrustes distance, which measures dissimilarity between matrices. The consensus configuration obtained from GPA allows for the visualization of emotional responses in a reduced dimensional space. This visualization facilitates the identification of key emotional drivers accounting for the differences between clusters, enhancing the interpretability of the results. Statistical analyses were computed using the software package XLSTAT (Lumivero, 2022) and Unscrambler (version 11, Camo).

## 3 Results

### 3.1 Steps 1 & 2: Concept development and refinement

The objectives of these two initial steps were to generate and refine initial ideas and themes for LB dish concepts based on adolescent preferences.

During the Jobs-To-Be-Done activities, adolescents highlighted several criteria for the new dishes intended for school-canteen use. These included the sensory JTBD aspects of being tasty, fresh, served immediately after preparation, properly cooked, with stronger flavours and contrasting textures. Additionally, emotional JTBD elements such as attractiveness, colourful presentation to evoke cheerfulness, novelty to inspire curiosity and surprise, and satiation for functional JTBD were emphasized. Adolescents also recognised the social role of the school canteen and suggested that dishes should accommodate their diverse personal tastes, such as by offering salt and spices for optional addition.

Results from the free-association-tasks derived from the three focus groups revealed significant variations in how they perceive the legumes, both in terms of sensory and emotional characteristics (Supplementary materials 1). Borlotti beans were associated with pleasant and tasty flavours, while cannellini beans evoked sadness. Peas were associated with bright colours, fava beans with vegetable gardens and seasonality and brown lentils were connected to occasions like New Year’s Eve and feelings of happiness. Chickpeas were associated with family and winter, while soybeans and soybean sprouts with ethnic characteristics. Red lentils were the least recognized and were mainly associated with sadness. The analysis of the focus group about dish creation led to the definition of 28 concepts of new LB dishes (Table 3), characterised by the use of a variety of ingredients, textures and cooking methods. These include salads and soups (cold and warm), crunchy elements (e.g., crispy parmesan wafer, mille-feuille with chickpeas) and legumes in different forms (e.g., soy spaghetti, cream of lentils/chickpeas).

### 3.2 Step 3: Concept validation

The refined concept of dishes developed during steps 1&2 (Table 3) were evaluated by the students based on their WTT them. The effect of “concept of dish” on WTT was significant (F_(27,2430)_ = 6.466, p < 0.0001, Cohen’s f = 0.268), with WTT mean values ranging from 4.27 to 6.58 across all concepts (Table 3). The effect of “subject” was significant with a large effect size indicating individual variability in the response (F_(90,2430)_ = 14.657, p < 0.0001, Cohen’s f = 0.737). Gender did not have a significant effect (F_(1,2492)_ = 1.841, p = 0.175) as well as the interaction gender x concept of dish (F_(27,2492)_ = 1.175, p = 0.244). FN level had a significant effect with a medium to large effect size (F_(1,2324)_ = 229.388, p < 0.0001, Cohen’s f = 0.314), with the LFN group being more willing to try the concepts compared to the HFN one (mean WTT = 6.10(2.25) vs 4.65(2.50) respectively). The interaction FN x “concept of dish” was largely not significant (F_(27,2324)_ = 1.118, p = 0.307).

### 3.3 Step 4: Concept selection & recipe development

**Fig. 2:**
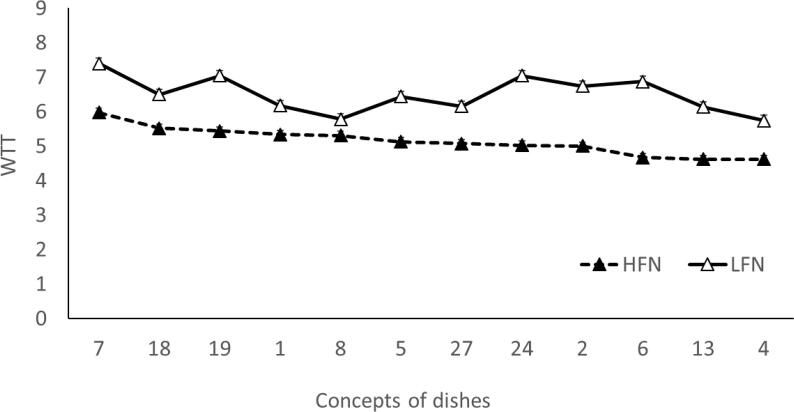
Willingness To Try (WTT) mean scores in Low and High Neophobic subjects (LFN; HFN) for the 12 selected concepts.

**Fig. 3:**
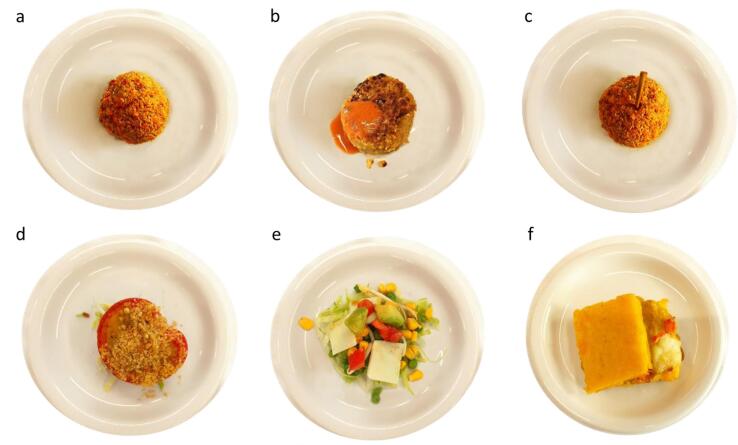
Photos of six LB dish samples prepared by the chefs; a) Lentil tomato arancino, b) Lentil-based burger, c) Lentil pumpkin arancino, d) Chickpea stuffed tomato, e) Peas and corn salad, f) Chickpea cream polenta sandwich.

Following concept validation, we aimed to choose the most promising dish concepts based on WTT for all the adolescents, considering different FN levels, and then to develop recipes. Twelve concepts were selected considering the best matching among the following results of the analysis of the WTT study: highest mean WTT across all subjects, concepts with mean WTT score above the group average in HFN and highest mean scores in LFN (Fig. 2). The twelve selected concepts of dishes were assessed against the five inclusion criteria (adherence to Mediterranean diet, sustainability, cost, preparation/serving time and feasibility) by a nutritionist and the chefs, with the five concepts that qualified all criteria being selected for preparation (Table 4). One additional concept was selected (concept 18) after substituting salmon with pumpkin, to enhance its sustainability. Chefs prepared recipes for the six concepts followed by preliminary assessment conducted by an internal panel comprising all chefs and the UniFi team for optimisation (Fig. 3). Changes included transforming the polenta sandwich into a crostino (concept 24), replacing avocado sauce with tomato sauce (concept 27), and replacing avocado and soy sauce with olive oil and orange (concept 6).

### 3.4 Step 5: Generation of dish specific sensory and emotional vocabulary

This step aimed to develop specific sensory and emotional descriptors for the dishes. Students evaluated the six prototypes through one-on-one interviews using the EmoSemio protocol (Spinelli and Jaeger, 2019, Spinelli and Monteleone, 2023, Spinelli et al., 2014). Employing a semiotic approach, the responses from the interviews were analysed, resulting in the creation of a participant-led lexicon (Table 5, Table 6). This lexicon was then used to measure sensory and emotional responses to dishes in a larger panel (A4) of adolescents. Based on the interviews, the salad dish was found to always be the lowest in the ranking. Furthermore, nutritional analysis indicated that the portion size should have been too large to fulfil the nutritional requirements for a meal in the school canteen. Thus, this sample was eliminated from the subsequent study, leading to five final dishes being evaluated by 138 adolescents (Lentil pumpkin arancino (LPA), Lentil tomato arancino (LTA), Lentil-based burger (LBK), Chickpea polenta crostino (CCP) and Tomato stuffed with chickpeas (STC).

### 3.5 Step 6: Prototype validation

The objective of this final step was to test the selected prototypes of the innovative dishes and gather comprehensive student feedback based on their liking, sensory and emotional responses.

#### 3.5.1 Mean liking scores for innovative dishes

The variation in mean liking among the innovative dishes was limited (from 52.95(23.93), to 59.63(24.18) and none of them was rejected (above “neither liked nor disliked”). However, a significant effect on liking mean scores of the factor “dish” was found (F_(4, 548)_ = 2.674, p = 0.031, Cohen’s f = 0.140), with the “arancino” samples LPA and LTA being more liked than the others, together with LBK. The sample LPA was liked significantly more than STC and CCP (Table 7). The effect of the factor “subject” was significant indicating individual variability in hedonic patterns with a very large effect size (F_(137, 548)_ = 4.772, p < 0.0001, Cohen’s f = 1.092).

**Table 7:** Two-Way ANOVA model (factors: dish and subjects) on liking and sensory responses. Mean scores and Standard Deviation (SD) for Lentil Pumpkin Arancino (LPA), Lentil Tomato Arancino (LTA), Lentil-Based Burger (LBK), Chickpea Polenta Crostino (CCP), and Chickpea Stuffed Tomato (STC). Number of respondents (n) and Cohen’s f (f) as a measure of the effect size is reported. Significance (p) of dish effect on sensory and liking responses to dishes. Significant sensory attributes (p < 0.05) are highlighted in bold. a,b,c letters indicate significant differences (p < 0.050) of means across dishes for each response as determined by Tukey's HSD (Honestly Significant Difference) Test.

	Dish												
	STC		CCP		LPA		LBK		LTA				
Response	Mean	SD	Mean	SD	Mean	SD	Mean	SD	Mean	SD	n	f	p
Liking	55.09ab	27.23	52.95 ab	24.18	59.63a	26.35	55.18 ab	24.46	58.11 ab	27.23	138	1.14	**0.031**
Sweet	2.28	1.30	2.34	1.48	2.52	1.52	2.27	1.30	2.18	1.29	134	0.12	0.139
Bitter	2.30a	1.58	2.01ab	1.44	1.70bc	1.25	2.04ab	1.54	1.61c	1.22	134	0.24	**0.000**
Sour	2.60a	1.59	2.51a	1.54	2.09b	1.39	2.51ab	1.72	2.34ab	1.39	134	0.16	**0.008**
Salty	3.03	1.57	2.75	1.38	2.71	1.48	2.81	1.48	2.82	1.40	134	0.11	0.185
Tasty	3.84a	1.80	3.41ab	1.78	3.44ab	1.83	3.33b	1.71	3.37b	1.76	134	0.16	**0.012**
Mild	3.09	1.67	2.94	1.72	2.96	1.60	2.91	1.60	2.75	1.43	134	0.09	0.396
Strong flavour	3.17a	1.69	2.96ab	1.62	2.49c	1.45	2.74bc	1.52	2.65bc	1.47	134	0.21	**0.000**
Spicy flavour	2.87a	1.69	2.65ab	1.61	2.54ab	1.41	2.35b	1.42	2.51ab	1.41	134	0.16	**0.011**
Fresh flavour	3.12	1.71	3.07	1.74	2.71	1.67	2.90	1.63	2.82	1.62	134	0.14	**0.044**
Contrasting flavours	3.39a	1.54	3.27a	1.59	2.76b	1.44	3.08ab	1.48	2.99ab	1.62	134	0.12	**0.000**
Melty	2.95	1.80	2.82	1.73	2.55	1.69	2.80	1.79	2.59	1.64	134	0.41	0.120
Crunchy	2.28bc	1.40	1.92c	1.29	3.21a	1.57	2.37b	1.58	2.92a	1.57	134	0.16	**0.000**
Soft	3.95	1.95	3.96	2.01	3.46	1.72	3.93	1.97	3.47	1.72	134	0.15	**0.008**
Firm	2.95b	1.54	3.24ab	1.73	3.38ab	1.67	3.07ab	1.63	3.41a	1.37	134	0.25	**0.018**
Dry	2.54bc	1.67	2.19c	1.53	3.14a	1.85	2.95ab	1.73	2.78ab	1.77	134	0.43	**0.000**
Grainy	2.84b	1.67	2.28c	1.42	3.81a	1.81	3.36a	1.89	3.63a	1.83	134	0.25	**0.000**
Hard	1.83b	1.30	1.92b	1.39	2.47a	1.45	2.17ab	1.55	2.53a	1.52	134	0.17	**0.000**
Creamy	2.87ab	1.67	3.05a	1.75	2.67ab	1.57	2.63ab	1.67	2.42b	1.35	134	0.13	**0.004**
Difficult to chew	2.05	1.49	1.76	1.09	1.98	1.36	1.70	1.03	2.00	1.42	134	0.39	0.050
Crumbling	2.35c	1.49	2.09c	1.56	3.45a	1.88	3.27ab	1.93	2.94b	1.70	134	0.47	**0.000**
Colourful	4.49a	1.65	4.05a	1.77	3.03b	1.59	3.24b	1.62	3.01b	1.49	134	0.13	**0.000**
Complex	2.94	1.62	2.77	1.51	2.57	1.56	2.85	1.66	2.53	1.48	134	0.12	0.057

The effect of gender on liking was significant (F_(1, 680)_ = 23.157, p < 0.0001; Cohen’s f = 0.185) with girls liking the dishes less than boys (mean liking = 49.8(25.49) vs 59.3(24.60), respectively). The effect of “dish” (F_(4, 680)_ = 1.489, p = 0.204) and the interaction “gender” x “dish” were not significant (F_(4, 680)_ = 0.210, p = 0.933). Food neophobia level was also significant with a medium effect size (F_(1, 615)_ = 50.877, p < 0.0001, Cohen’s f = 0.288), with the LFN group liking the dishes more compared to the HFN one (mean liking = 62.6(23.02) vs 48.7(25.78) respectively). Again, the effect of “dish” (F_(4, 615)_ = 1.565, p = 0.182) interaction “FN” x “dish” was not found to be significant (F_(4, 615)_ = 1.050, p = 0.381).

#### 3.5.2 Sensory properties of the innovative dishes

The products significantly differed for the variables *bitter, sour, tasty, strong, spicy, fresh, contrasting, crunchy, soft, firm, dry, grainy, hard, creamy, crumbling* and *colourful*, but not for the variables s*weet, salty, mild and melty,* with a large effect size for *crumbling, dry* and *melting* (Table 7). Differences in *complexity* were close to significance (F_(4,527)_ = 2.307, p = 0.057).

**Fig. 4:**
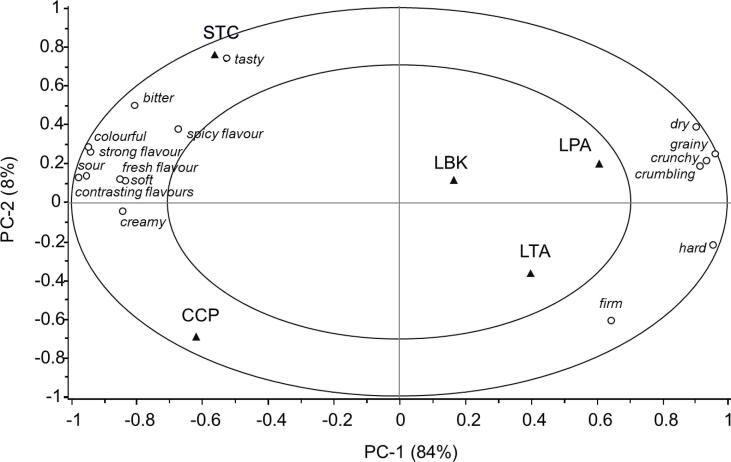
Sensory differences and similarities among dishes (Lentil pumpkin arancino (LPA), Lentil tomato arancino (LTA), Lentil-based burger (LBK), Tomato stuffed with chickpeas (STC), Chickpea polenta crostino (CCP): correlation loading plot from Principal Component Analysis computed on panel averages of each significant attribute (p < 0.050) based on the two-way ANOVA model. Samples were included as dummy variables. Outer and inner circles on the map represent 100 % and 50 % explained variance respectively.

Results of the PCA computed on the significant sensory attributes are summarized in the correlation loading plot in Fig. 4. The first two significant dimensions of the perceptual map account for 92 % of the variation. Along the first component, from the right to the left, samples LPA, LTA and LBK are separated from STC and CCP. This component is positively associated with the descriptors *dry, grainy, crunchy, crumbling*, *hard* and *firm*, and negatively associated with *soft, creamy, tasty, bitter, sour, contrasting, spicy* and *strong flavours*, *colourful* and *fresh* attributes. The second component contributes to further discriminating STC from CCP mainly in relation to the “*tasty*” attribute, with STC being described as tastier. All sensory variables were well related to the PCs, being located very close to the outer ring that indicates 100 % explained variance.

#### 3.5.3 Sensory drivers of liking and individual differences in hedonic patterns

**Fig. 5:**
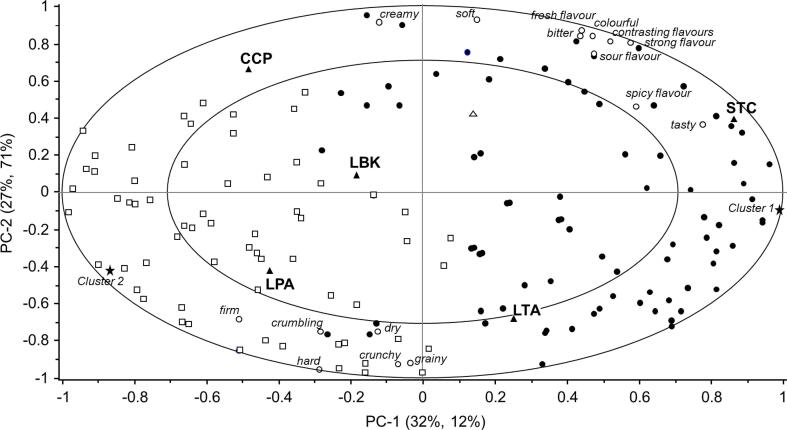
Internal Preference Map: correlation loading plot from Principal component regression (PCR) on the first two dimensions (PCs 1 and 2). Coordinates: liking data (X) and sensory properties (Y) of samples (LPA, LTA, LBK, STC, CCP). = Adolescents of Cluster 1; = Adolescents of Cluster 2; = sensory variables = Cluster 1 and 2 mean liking score. Samples were included as dummy variables. Outer and inner circles on the map represent 100 % and 50 % explained variance respectively.

To better explore liking patterns considering individual differences and to identify the sensory drivers of liking between participants, a PCR (Internal Preference Map) was computed on the liking data (X) and sensory data (Y) for the five dishes (Fig. 5). In the IPM map, the first two components represented 32 % and 27 % of the explained variances in X, and 12 % and 71 % in Y. All sensory variables were well related to the PCs, being located very close to the outer ring that indicates 100 % explained variance. Along the first dimension STC and LTA are well separated from CCP, LPA and LBK, whereas along the second dimension CCP and STC are well separated from LPA and LTA. The map shows that subjects are spread along the two dimensions indicating that adolescents do not share a common pattern of liking for the dishes. A good separation of adolescents' liking for the dishes was found when looking at the two segments, identified through AHC (cluster 1, n = 74; cluster 2, n = 64) on liking data. Cluster 1, located on the right side of the PC1 (black circles), showed a preference for the tomato-based dishes (STC and LTA); its preference was mainly driven by *fresh, bitter, sour, strong, spicy, tasty* and *contrasting* flavour and *colourful* appearance. This segment also showed a lower preference for CCP, LPA and LBK dishes driven by *firm, crumbling* and *hard* texture attributes. On the other hand, most adolescents belonging to cluster 2 were located on the negative side of PC1 (white squares). Drivers of liking and disliking of cluster 2 were the same but obviously with an opposite sign of those described for cluster 1. The two clusters are widely spread along PC2.

**Fig. 6:**
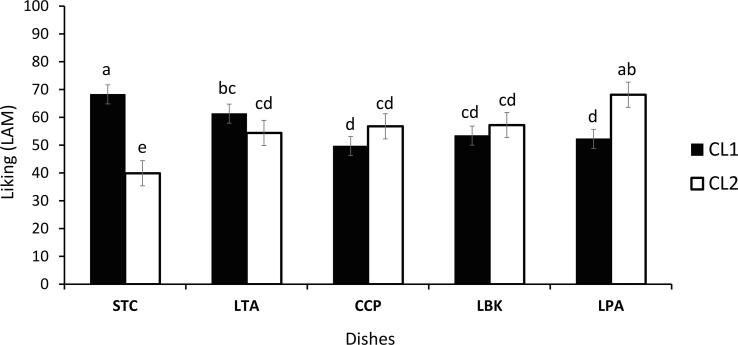
Two Way ANOVA: representation of the cluster x dish interaction effect. Mean values and LSD 95 % significant differences (letters).

When examining liking for the dishes using a Two-way ANOVA model, no direct effect of “cluster” (F_(1,680)_ = 0.859, p = 0.354) or of “dish” were observed (F_(4,680)_ = 1.666, p = 0.156). Nevertheless, a significant interaction effect between “cluster” and “dish” indicated differential liking patterns for specific dishes among the clusters (F_(4,680)_ = 16.973, p < 0.0001), with cluster 1 liking STC more and LPA less than cluster 2, further confirming and expanding the results of the cluster analysis. The effect size of the interaction is moderate (Cohen’s f = 0.316). Significant differences in liking among clusters for all samples are reported in Fig. 6. Cluster 1 found STC pleasant, whereas cluster 2 rated it only slightly unpleasant. On the other hand, LPA was rated as pleasant by cluster 2 and as neither liked nor disliked by cluster 1. Indeed, LPA and all dishes with the exception of STC were liked by cluster 2, whereas STC and LTA were the most liked dishes by cluster 1. No difference in liking for LTA, CCP and LBK was found between clusters, which rated the dishes between “neither like nor dislike” and “like slightly”.

#### 3.5.4 Cluster characterisation

While the clusters did not differ in gender distribution (p = 0.517), the One-way ANOVA revealed that the clusters significantly differed in their food neophobia score (F_(1,683)_ = 8.290, p = 0.004), with cluster 2 reporting a higher mean neophobic score than cluster 1.

#### 3.5.5 Emotional responses to the dishes

**Fig. 7:**
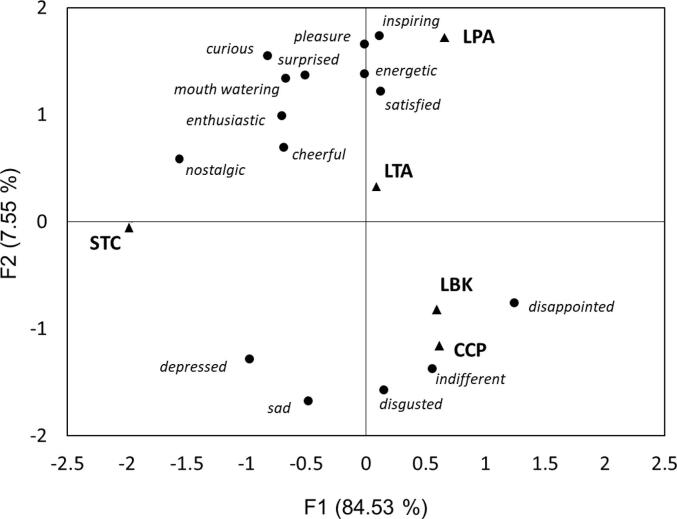
Generalized Procrustes Analysis: Consensus Space on emotions configuration from Cluster 1 and Cluster 2.

To investigate the emotional responses associated with the dishes, we looked at the Consensus Space on emotions configuration of cluster 1 and cluster 2 after a GPA (Fig. 7). Considering sample differences resulting from emotional data, simply averaging the results across all respondents would have overlooked the two distinct clusters we previously identified based on liking responses to prototypes. The potential emotional differences among the clusters justify the use of GPA.

The first two dimensions of the map accounted for 92.08 % of the systematic variation. PC1 was positively associated with the emotion of *disappointment*, whereas it was negatively associated with the emotion of *nostalgia*. PC2 was positively associated with a positive experience (*satisfied, surprising, energetic, pleasant, inspiring, mouth-watering, curious, enthusiastic, happy, nostalgic*) and negatively associated with negative (*depressed, sad, disappointed, disgusted*) or neutral (*indifferent*) experience. PC1 separated the dishes LBK, CCP, associated with *disappointment*, from STC, which was found to be the most *nostalgic* dish. The second dimension showed a good separation of LPA, associated with positive emotional responses, high in arousal, such as *inspiring, energetic, pleasant, curious* and *surprise* from LBK and CCP, which were associated with negative emotional responses such as *depressed, sad* and *disgusted*. The dish LTA was also associated with positive emotions, even if to a lower extent compared to LPA. Results from the analysis of liking data and emotion measurements sustain the conclusion that both arancini, LPA and LTA, could be included in the school canteen menu as they fulfil, more than the others, the JTBD identified in step 1.

## 4 Discussion

This study aimed to develop new, healthy, and widely accepted LB dishes for school canteens by using a co-creation approach with adolescents in a low socioeconomic status area. This approach aimed to explore the determinants of acceptance among adolescents, such as liking and emotional responses, and to incorporate these findings into the development process to ensure the dishes meet their preferences and needs. Recognising adolescents’ right to a healthy diet within planetary boundaries and involving them in the food systems transformation is long overdue (EAT & UNICEF, 2020). Successfully promoting healthy and sustainable eating to adolescents requires acknowledging their unique development trajectory, social context and their food-related roles and needs (Neufeld et al., 2022). Engaging adolescents in the innovation process of LB dishes is a promising strategy, as co-creation activities can result in liked and accepted tailor-made products. In our study, we worked together with students to co-create new LB dishes tailored to their preferences. The characteristics of the selected school in an area of lower SES and higher rate of immigration (2nd and 3rd generation) allowed a more inclusive approach by engaging students from diverse backgrounds. In this way, we aimed to ensure that their unique perspectives and different culinary traditions were thoroughly integrated into the dish development process. Our approach recognises the significance of tackling social inequalities linked to obesity and overweight among adolescents. To do so, we used the participatory approach combination of co-creation, with a set of methods. Prior research has highlighted the effectiveness of focus group discussions (Galler et al., 2022) and the SCAMPER technique (Boonpracha, 2023) in generating ideas and facilitating brainstorming activities. The Jobs-To-Be-Done approach was found effective in identifying needs and unfulfilled desires of adolescents related to the experience of eating in school canteens. Twenty-eight innovative dish concepts were co-created with adolescents and the contribution of chefs. These concepts varied across multiple dimensions, including the type of legumes utilized (soybeans, soybean sprout, chickpeas, peas), the variety of dishes (ranging from fresh salads to sandwiches and soups), diverse sensory characteristics (creamy, colourful, crunchy, contrasting flavours) and serving temperatures (warm and cold). This diversity underscores the success of our methods in sparking creativity and innovation.

A contributing factor to the high variety of concepts was the multicultural environment of the school, which facilitated the participation of students from diverse ethnicities, such as Asians, in the brainstorming sessions. This diversity in perspectives led to a greater variety of ingredients being used in the recipes, including soybean sprout, commonly consumed in Asian cuisine (Dhakal et al., 2014). Combining these ingredients together with those traditional Italian ones, like peas, resulted in recipes with a higher novelty degree, while retaining a sense of familiarity—a factor strongly associated with liking in adolescence (Dinnella et al., 2016).

This variety and diversity in ingredients and cooking methods employed in the conceptualization of dishes can be also attributed to the involvement of professional chefs. To ensure the healthiness and palatability of the final dishes, we engaged four chefs in the co-creation activities, both in the concept identification and recipe development stages. This was instrumental in ensuring that the concepts generated during the brainstorming sessions with students aligned with our objectives of promoting healthy eating. While students were tasked with brainstorming healthy dishes, it was conceivable that some suggestions might have included cooking methods that compromised the nutritional quality, such as frying. In such instances, the expertise of the chefs came into play, as they devised alternative cooking techniques to uphold the nutritional value of the final recipes. Moreover, the free-association-tasks, during which students reported that they associate certain legumes with negative and others with positive sensory and emotional characteristics, were particularly important, as chefs took these associations into consideration when developing the final recipes, ensuring that the dishes resonated with the students' sensory and emotional preferences while maintaining their nutritional integrity.

For a new food to be appreciated, it should incorporate elements of both novelty and familiarity (Berlyne, 1970, Köster and Mojet, 2007). This means that it should be surprising, to attract the interest and engage the subjects, but also somehow reassuring (for example including some aspect of familiarity). This is particularly important for food neophobic adolescents, as food neophobia served as a well-known barrier to new product acceptance. While it is generally expected to decrease in late adolescence and adulthood, it remained relatively high in our sample, with mean scores 19.79 for the sample in step 3 and 21.32 for the sample in step 6, and median scores of 20 for step 3 and 21 for step 6. These scores are similar to those found in children of different European countries aged 9–12 years (Italy, Spain, Finland, Sweden, and UK) (Laureati et al., 2015, Proserpio et al., 2020). The similarity in food neophobia scores across these diverse regions highlights that high levels of food neophobia are not unique to Italy but are a common challenge in different dietary contexts. An innovative aspect of our approach was that the food innovation process incorporated the needs and tastes of adolescents higher and lower in FN, with the objective of developing and selecting new dishes that could be accepted by all.

Consistent with existing literature (Karaağaç and Bellikci-Koyu, 2023, Predieri et al., 2020), we observed a negative association between FN and WTT, as well as liking for the dishes, with high neophobic adolescents expressing lower WTT and liking scores, for both dish concepts and actual dishes (p < 0.0001 respectively). However, mean WTT scores for dish concepts among highly neophobic subjects were close to the scale mean. Likewise, our liking data indicate that all the five dishes were well received (mean liking = 56.2(25.30)) even among the high neophobic adolescents, who rated them as “neither liked nor disliked” (mean liking = 48.7(25.78)). These findings suggest that our co-creation approach partially overcame the FN barrier resulting in novel dishes that were not rejected by this demographic. This is particularly significant as FN typically serves as a barrier to consuming novel foods and reduces dietary variety (Karaağaç & Bellikci-Koyu, 2023).

The IPM facilitated the segmentation and comparison of students based on their liking, allowing for the identification of samples that mitigate the disliking among the HFN subjects while still being well-appreciated by those with lower levels of neophobia (e.g., sample LTA). Moreover, the IPM clearly showed that appearance, texture and flavour significantly influence adolescents’ liking, with attributes such as *colourful* appearance, *tasty, strong*, *contrasting* and *fresh* flavours and *crunchy, crumbling* and *hard* textures being key drivers of preference. The IPM also revealed individual differences among adolescents, particularly evident in cluster 2, which exhibited a higher mean neophobic score. This segment showed a preference for certain texture attributes (e.g., *firm*), and a decreased liking for sensations like *bitterness, sourness, strong* flavours and *spicy* notes*,* aligning with previous research (Appleton et al., 2019). Rich flavour (e.g., *tasty*, *spicy*) and appearance (e.g., *colourful*) emerged as the main drivers of liking for cluster 1. This is consistent with prior studies that link colour intensity with hedonic responses, as well as associations between complex textures like crunchiness and higher preference among adolescents (Dinnella et al., 2016).

To grasp the challenges and requirements students encounter with school canteen food, we employed the JTBD approach seeking to enhance its acceptability. Students reported that the dishes served in school canteens should fulfil various sensory, emotional, social and functional “jobs”: they should be attractive, tasty, fresh, served immediately, well cooked, with stronger flavours and contrasting textures, colourful, satiating, more elaborated and with an element of novelty. Upon reviewing the sensory characteristics of the final dishes —marked by *freshness, tasty, strong*, and *contrasting* flavours, *colourful* attributes and a variety of textures (*soft, hard, crumbling* etc.)— our co-creation approach was able to get the “jobs” done. Analysis of the consensus plot revealed that two of the new dishes (LPA and, to a lower extent, LTA) evoked positive emotional responses high in arousal and novelty (*inspiring, satisfying, surprising, curious, energetic*) among all adolescents, indicating their alignment with the emotional JTBD identified in step 1.

Studying the emotional responses to the dishes provided valuable insights beyond mere liking, discriminating the dishes more effectively for their emotional profile, and therefore facilitating a more nuanced understanding of product acceptance and adoption. As Köster (2003, p.362) clearly indicated, assuming that the most liked prototype will be the one successful in the market is a mistake. When dealing with new products it is much better to select “a slightly less liked, but somewhat more complex product […] that, in becoming at the same time somewhat less complex through exposure, will meet the consumer half way”. High arousal emotions are inherently related to perceived complexity (Spinelli et al., 2018) and thus their evaluation should be always considered in validating co-created products.

The GPA allowed us to obtain a consensus between the clusters on the emotional responses, providing a comprehensive understanding of how different groups of adolescents perceive the dishes. Specifically, emotional characterization revealed that the LPA dish elicited positive valence emotions such as *inspiring, satisfied*, *pleasant* along with positive valence high arousal emotions such as *energetic* and *surprise*. In contrast, dishes like LBK and CCP were associated with negative valence high arousal emotional responses such as *disgust,* negative valence low arousal emotions such as *sadness* and *indifference,* indicating potential challenges in their acceptance. Notably, LPA's association with high arousal emotions, particularly *surprise*, suggests its higher novelty degree compared to other dishes, without being disliked by more neophobic adolescents. This makes this dish a good candidate for the introduction in the school canteens and further highlights the success of our co-creation methodology in developing novel and accepted LB dishes.

Our findings indicate that gender did not influence the WTT the dish concepts, suggesting that both boys and girls were equally open to trying the proposed dishes. This equitable reception may be attributed to the inclusive co-creation process, which incorporated inputs from both genders. However, girls demonstrated a lower overall liking for the final dishes, potentially reflecting differing sensory expectations compared to boys. Nonetheless, it is noteworthy that girls did not reject the dishes (mean liking = 49.84(25.49)).

While our study did not directly test the acceptance of the developed PB dishes in real school canteen conditions, particularly against other menu options, we found that their co-creation with adolescents inherently increased their likelihood of acceptance. By involving adolescents in the dish development process, we ensured that the dishes reflected their preferences and priorities (Jobs-To-Be-Done). Therefore, we anticipate that these dishes would be well-received by a broader adolescent population in Italy, as the co-creation process incorporated food aspects important to this demographic. Further research should validate the feasibility and desirability of these dishes in school settings, also considering repeated exposure to promote healthier eating habits.

Our findings contribute to the literature on co-creation and adolescent nutrition by providing evidence that participatory methods can lead to the development of preferred and accepted food products. This study underscores the importance of considering the unique preferences and needs of adolescents in nutritional interventions, enriching the theoretical framework surrounding co-creation and health promotion.

### 4.1 Limitations

This study comes with some limitations, as dish evaluation occurred in a school environment rather than a controlled sensory lab. While this is advantageous because it provides a more natural setting, putting adolescents more at ease compared to the lab setting, it also results in lower control over sources of variability. Although interactions between students were minimized and supervised, some may have occurred. However, it is unlikely that these interactions significantly impacted the results. Conducting the study in a more natural setting enhances ecological validity despite this limitation. Additionally, students attending a hospitality-focused school may possess greater knowledge and sensitivity to food choices, potentially influencing results. While this expertise is beneficial during co-creation sessions, we also found that food neophobia was relatively high among the students, with similar levels to those found in pre-adolescents in other studies (Proserpio et al., 2020). This supports the idea that the sample was not biased by a higher involvement with food. Furthermore, while the school accommodates many students from lower socioeconomic backgrounds and different ethnicities, individual SES and ethnicity data were not collected, limiting our ability to study the effect of these variables on the acceptance of new legume-based foods. Additionally, while direct measures of SES were not collected, the recruitment strategy significantly reduces the likelihood of a skewed sample. The inclusion of almost all students in the class means that the diversity within the low SES population was likely captured, providing a robust basis for our findings.

### 4.2 Future research and implications

This study opens several avenues for future research. Research could explore the applicability of this co-creation approach across different socioeconomic and socio-cultural contexts to determine its effectiveness in diverse populations. From a practical perspective, the successful co-creation of LB dishes highlights the potential for involving adolescents in the design of school meals to increase acceptance and consumption of healthy foods. These results further support the recommendations of European institutions, such as the European Commission, that suggest that schools, food procurement services and policymakers should consider integrating co-creation methodologies into their food service programs to foster healthier eating habits among students and reduce food waste (Quaglia & Guimaraes Pereira, 2021). This research has identified many sensory characteristics of dishes increasing the likelihood of acceptance (e.g., colourful presentation, varied textures, strong/appealing flavours). Emotional characterization can guide the development of new products that not only meet sensory expectations but also resonate on an emotional level, potentially leading to higher acceptance and consumption rates. These results can be seen as a first stepping-stone to providing guidance for canteen managers and food services in developing new PB dishes that can be accepted by everybody, including the adolescents higher in food neophobia. Furthermore, the co-creation methodology used in this study can be adapted and simplified to be applied in other settings, such as hospitals, where involving patients in the design of meals could lead to improved patient satisfaction and health outcomes. In general, translating research into practice often requires a reduction of complexity, and this approach can be tailored to fit the specific needs and constraints of different organizations and populations, ultimately leading to more effective and user-centered solutions.

## 5 Conclusion

This study offers valuable insights into adolescent nutrition and food innovation, particularly in school canteen settings. By employing co-creation methodologies, including the Jobs-To-Be-Done approach, this research developed sustainable and healthy LB dishes tailored to adolescents' preferences, considering the needs and tastes of adolescents with different levels of food neophobia. By involving adolescents from diverse backgrounds, the study addressed social inequalities and advanced theoretical understanding by demonstrating how co-creation processes can overcome barriers to PB food consumption, such as FN, among adolescents. By considering individual differences not only in liking but also in emotional responses, the study highlights the importance of integrating emotions into the innovation process, from the early stage of idea generation to the validation of the prototype.

Overall, this research contributes to the growing body of literature on co-creation in food innovation and underscores the importance of incorporating diverse perspectives and understanding individual differences in designing sustainable and appealing food solutions for adolescents. By bridging theory and practice, the study provides actionable insights for stakeholders involved in adolescent nutrition, school food programs, and food product development.

## CRediT authorship contribution statement

**Kokkorou Margarita:** Writing – original draft, Visualization, Investigation, Data curation, Formal analysis. **Spinelli Sara:** Writing – review & editing, Methodology, Investigation, Formal analysis, Conceptualization. **Dinnella Caterina:** Writing – review & editing, Methodology, Investigation, Conceptualization. **Pierguidi Lapo:** Writing – review & editing, Investigation. **Wollgast Jan:** Writing – review & editing. **Maragkoudakis Petros:** Writing – review & editing. **Monteleone Erminio:** Writing – review & editing, Visualization, Methodology, Funding acquisition, Formal analysis, Conceptualization.

## Declaration of competing interest

The authors declare that they have no known competing financial interests or personal relationships that could have appeared to influence the work reported in this paper.

## Data Availability

Data will be made available on request.
