# Supplementary material for: Co-creating innovative and accepted legume-based dishes for school canteens with adolescents in a low socioeconomic area
Source: Food Qual Prefer. 2025 Feb;123:105343. doi: 10.1016/j.foodqual.2024.105343 (PMC11591174; doi:10.1016/j.foodqual.2024.105343)
Supplement: Supplementary Data 2 [file mmc2.pptx]

## Slide 1
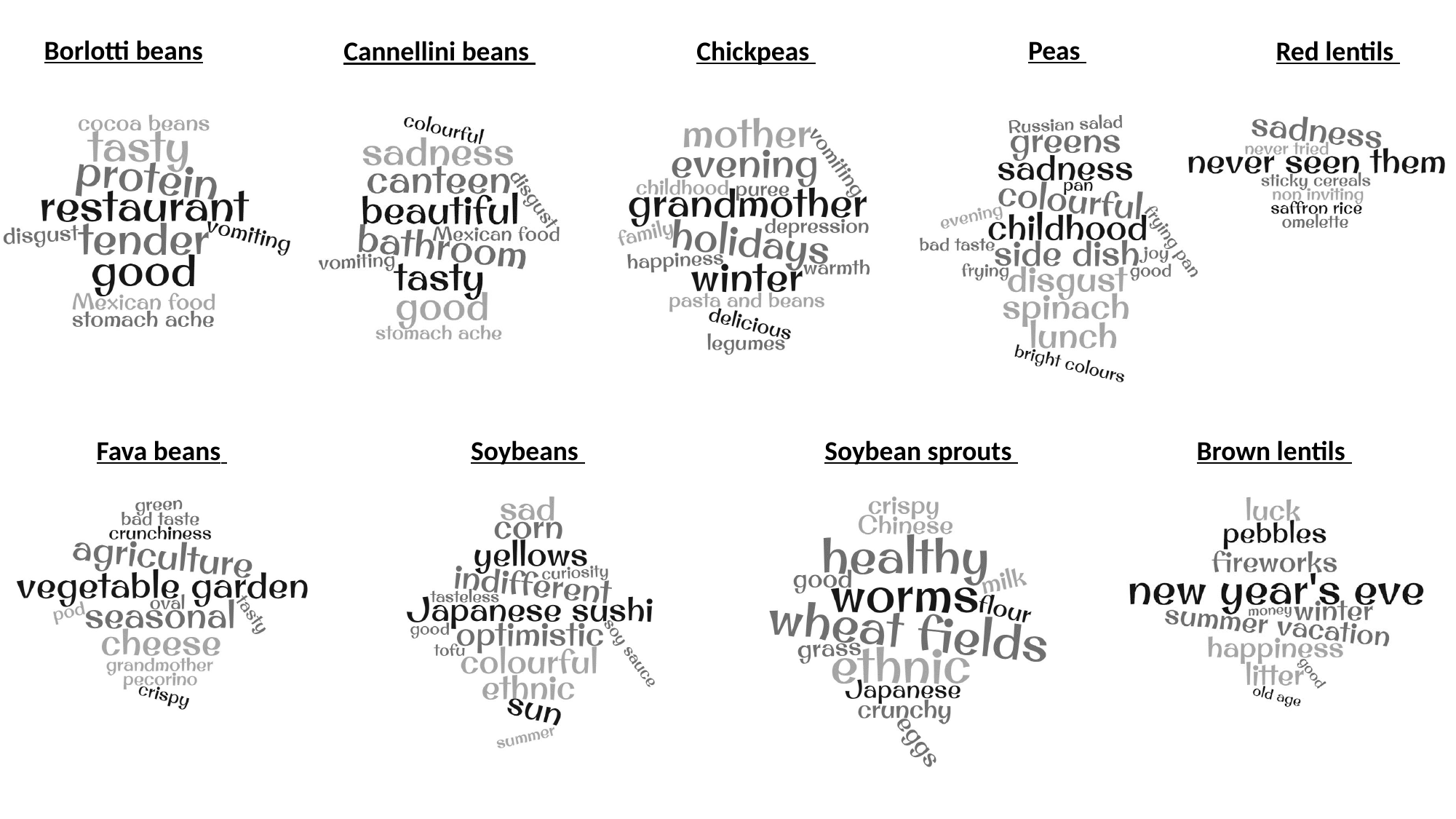

Borlotti beans
Peas
Chickpeas
Red lentils
Cannellini beans
Fava beans
Soybeans
Soybean sprouts
Brown lentils
